# Wound Dehiscence After Occipital Encephalocele Surgical Repair in a Neonate: Management Alternative

**DOI:** 10.7759/cureus.23685

**Published:** 2022-03-31

**Authors:** Juan P Navarro-Garcia de Llano, Aurelio Ponce-Ayala, Alejandro Ceja-Espinosa, Carlos D Vera-Márquez, Rafael Mendizabal-Guerra

**Affiliations:** 1 Vascular Neurosurgery, Instituto Nacional de Neurología y Neurocirugía, Mexico City, MEX; 2 Neurosurgery, Hospital Juarez De Mexico, Mexico City, MEX; 3 Neurosurgery, Hospital Juarez de Mexico, Mexico City, MEX

**Keywords:** wound dehiscence, occipital encephalocele, pediatrics neurosurgery, encephalocele, delayed wound healing

## Abstract

Encephaloceles are congenital malformations of the neural tube, mostly located in the occipital region in the Western world. Its presence is related to many complications, among which cognitive impairment and death are the most important. The diagnosis is usually made in the prenatal period, but sometimes due to poor control, this is not feasible. Surgery is required as early as possible to prevent further damage. Sometimes we can face complications related to the procedure, such as wound dehiscence, which has been the aim of this work. Many different types of treatments have been proposed for this complication: nevertheless, they result in invasive management. We present the case of a neonate's wound dehiscence, managed with potable water washes and a correct sterile technique, shown to be safe, reduce the in-patient costs, as well as improve the patient’s and their family's quality of life (QoL).

## Introduction

Occipital encephaloceles are congenital malformations of the neural tube characterized by a fusion defect of the skull bones, resulting in a space through which structures such as meninges, CSF, and brain tissue can herniate. An incidence of 1-4 cases per 10,000 live births is reported, making the occipital defect the most common in the Western world, accounting for up to 85% of reported cases [1,2].

Ideally, the diagnosis is made in the prenatal period using ultrasound devices. This allows early identification of the defect, reducing the morbidity and mortality that this pathology represents [2]. In developing countries, this can be challenging due to social problems related to education and health center availability. Thus, a large sector of the population does not have proper prenatal care. This results in a late diagnosis with a slight increase in the risk of morbidity and mortality. However, an adequate approach with imaging studies (MRI or CT) and early correction can decrease the incidence of complications such as cognitive impairment [1].

Different surgical techniques have been established for defect repair, and we can divide them into two large groups: those performed during the prenatal period (fetal surgery) and those after birth [2].

Dehiscence can be defined as the loss of wound integrity that occurs at least two weeks after the surgical procedure. It is a complication that is commonly related to infection of the surgical site. However, many other causes could explain it, such as malnutrition, multiple surgeries on the same site, previous irradiation, coagulation disorders, etc., and we would not necessarily require antibiotics to manage it [3].

Here, we present a case and explain the management given to a neonate who underwent occipital encephalocele repair and got complicated with wound dehiscence.

## Case presentation

**Figure 1:**
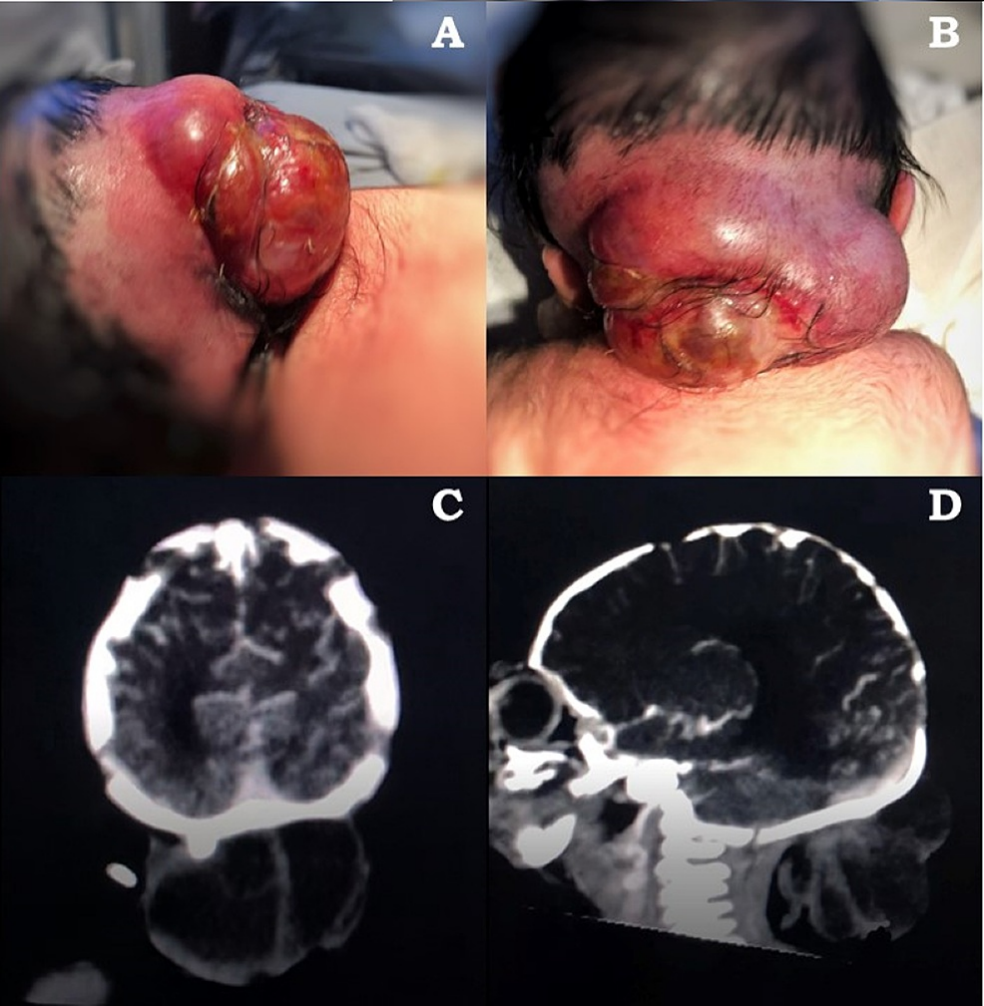
Occipital encephalocele (A) Occipital encephalocele lateral view, (B) occipital encephalocele superior view, (C) axial head CT showing the cystic protrusion consistent with occipital encephalocele, and (D) sagittal head CT image showing the same cystic protrusion with heterogeneous densities.

We present the case of a one-day-old neonate who is the product of a 38-week pregnancy with poor control. After natural childbirth, the pediatrician detected a defect at the occipital level, with an irregular, heterogenous, and depressible mass protrusion (Figure 1A). The patient then underwent head CT, observing a relatively small bone defect at the subtorcular level with a non-enhancing cystic sac whose content density was heterogeneous, making the diagnosis of encephalocele (Figure 1B-1D). 

Surgical resection was then performed using the technique described in Pediatric Neurosurgery by Goodrich [4]. Incising horizontally on the skin and completing it with scissors, the posterior arachnoid layers were opened using forceps to identify the abnormal neural tissue, which was resected using cautious bipolar electrocoagulation and scissors. Then, the periosteum was incised and reflected with a periosteal elevator, followed by dura closure with absorbable suture and cutaneous tissue remodeling.

**Figure 2:**
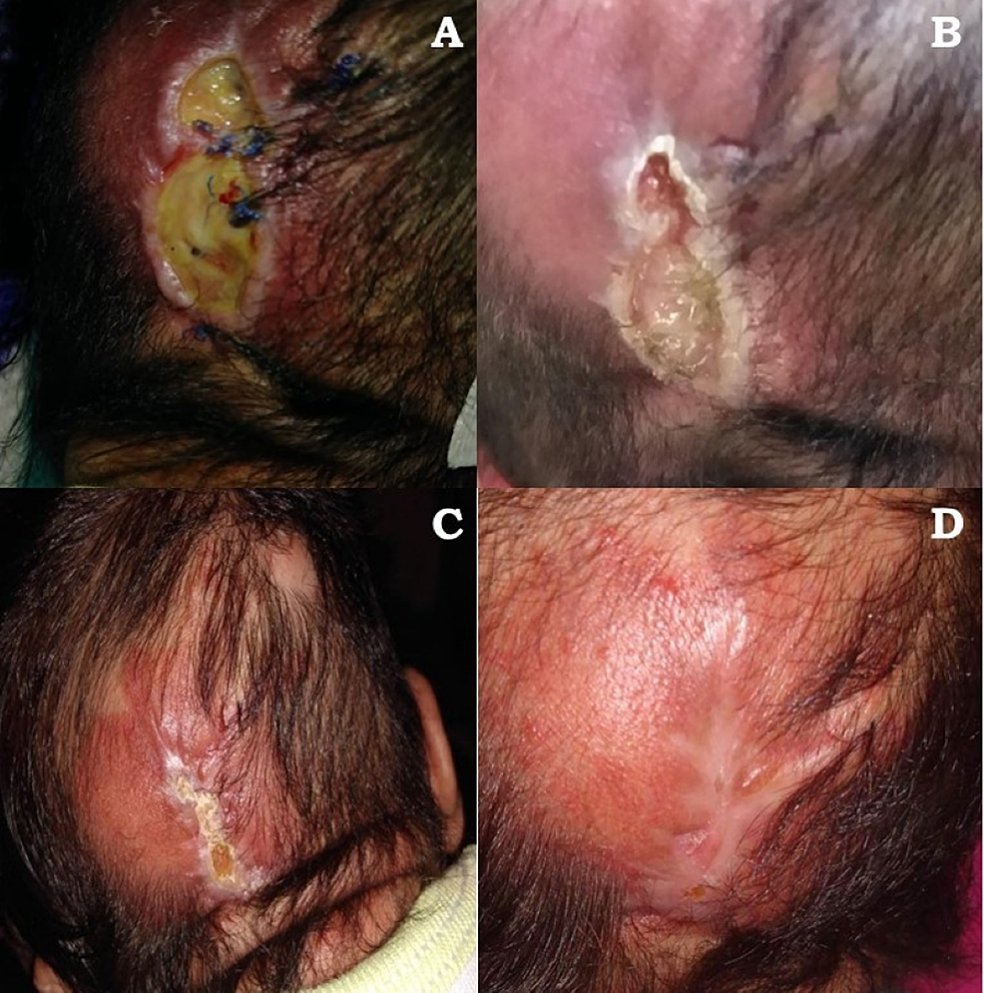
Wound dehiscence (A) Wound dehiscence 18 days after the surgical procedure, (B) wound healing process five days after initial management with potable water, (C) 15th day of treatment, and (d) monthly visit showing complete wound healing and scaring.

On the eighth postoperative day, the wound started to open with a serous secretion, shortly after we lost patient follow-up for the next ten days. When the patient returned, complete wound dehiscence was observed (Figure 2A). Despite this, no evidence of infection was found. Due to having recently undergone serious hemodynamic difficulties during the previous surgical procedure, more conservative therapy was then started with potable water washes and posterior application of *Triticum vulgare* extract (Italdermol®) three times a day for the next 30 days in the out-patient environment. Five days later, a marked improvement was observed (Figure 2B), and by day 15, it was completely closed (Figure 2C). At the next monthly visit, the patient presented with complete recovery and correct wound healing (Figure 2D).

## Discussion

Encephalocele surgical repair is a procedure that can be challenging for a multidisciplinary team and is related to different complications such as CSF leak, hydrocephalus, meningitis with possible progression to septic shock, and wound dehiscence [1].

Worldwide, the management of wound dehiscence commonly follows a few accepted steps. First, a wound swab culture should be obtained, followed by debridement of non-viable tissue and systemic broad-spectrum antimicrobial therapy for a long period (four to six weeks) [3]. However, the risk of developing antimicrobial resistance, necrotizing enterocolitis, late-onset sepsis, and death related to long-term use of broad-spectrum antibiotics has been described before [5].

Other therapies have already been proposed for the management of this complication, such as the use of negative pressure wound therapy, among which we can find vacuum-assisted closure (VAC) devices in charge of bringing the wound margins together, cleaning, and promoting the formation of granulation tissue, thus helping to reduce the need for painful dressing changes, healing time, and allowing the patients an earlier return to their daily activities [3]. Despite all these benefits, VAC still represents a high-cost therapy with an average daily cost of $94.01 [6]. This was the main reason that made us look for a safe and straightforward alternative, useful in underserved areas around the globe.

When the patient came back to us, we intentionally searched for signs of infection. As we did not find any, we decided to start daily wound washes with potable water, always following a sterile wound management technique and avoiding unnecessary antibiotics that could have ended in something worse. We demonstrated that correct control of surgical wound dehiscence could be done without the need for antibiotic administration or without performing invasive procedures.

## Conclusions

In our experience, the daily use of potable water as the management for wound dehiscence had a great outcome and reduced the in-patient costs compared to other therapies. We avoided unnecessary antibiotics and prevented the patient from undergoing another surgical procedure, which could have been important physical stressor for the patient and a psychological burden, especially for the parents. We consider that this conservative technique should be promoted, although not generalized, as each patient should be evaluated separately and receive adequate therapy depending on many factors.

We consider that more studies and social advances aiming the follow-up inconsistency should be created to reduce the incidence of this type of complication in the first place.
